# Adherence to European Clinical Practice Guidelines for Secondary Prevention of Cardiovascular Disease: A Cohort Study

**DOI:** 10.3390/ijerph15061233

**Published:** 2018-06-11

**Authors:** Josep Maria Pepió Vilaubí, Domingo Orozco-Beltrán, Alessandra Queiroga Gonçalves, Dolors Rodriguez Cumplido, Carina Aguilar Martin, Adriana Lopez-Pineda, Vicente F. Gil-Guillen, Jose A. Quesada, Concepcion Carratala-Munuera

**Affiliations:** 1 Equip d’Atenció Primària Tortosa Oest, Institut Català de la Salut, 43500 Tortosa, Tarragona, Spain; jmpepio.ebre.ics@gencat.cat; 2 Chair of Family Medicine, Miguel Hernandez University, 03550 San Juan de Alicante, Spain; adriannalp@hotmail.com (A.L.-P.); vte.gil@gmail.com (V.F.G.-G.); jquesada@umh.es (J.A.Q.); atencion.primaria@umh.es (C.C.-M.); 3 Unitat de Suport a la Recerca Terres de l’Ebre, Institut Universitari d’Investigació en Atenció Primària (IDIAP) Jordi Gol, 43500 Tortosa, Tarragona, Spain; aqueiroga.ebre.ics@gencat.cat (A.Q.G.); caguilar.ebre.ics@gencat.cat (C.A.M.); 4 Unitat Docent de Medicina de Família i Comunitària Tortosa-Terres de l’Ebre, Institut Català de la Salut, 43500 Tortosa, Tarragona, Spain; 5 Fundació Institut Català de Farmacologia, Universitat Autònoma de Barcelona, 08035 Barcelona, Spain; dr@icf.uab.cat; 6 Unitat d’Avaluació, Direcció d’Atenció Primària Terres de l’Ebre, Institut Català de la Salut, 43500 Tortosa, Tarragona, Spain

**Keywords:** cardiovascular disease, secondary prevention, primary health care, health systems

## Abstract

To provide a better understanding of the actions taken within health systems and their results, this study aims to assess clinicians’ adherence to clinical practice guidelines (CPGs) regarding recommended treatments in patients with cardiovascular disease in primary care settings, and to determine the associated factors. We conducted an ambispective cohort study in 21 primary care centres in 8 Spanish regions. Patients diagnosed with coronary heart disease, stroke and/or peripheral arterial disease were included. Patients who received the treatment recommended in the European guidelines on cardiovascular disease prevention (CPG’s adherent group) were compared with patients who did not (CPG’s non-adherent group). The outcome variables were cardiovascular hospital admissions, all-cause and cardiovascular mortality during follow-up. Of the 438 participants, 38.6% (*n* = 169) received the drug therapies recommended in the guidelines. The factors that increased the likelihood of good adherence to CPG’s were being diagnosed with hypertension (*p* = 0.001), dyslipidaemia (*p* < 0.001) or diabetes (*p* = 0.001), and not having a psychiatric disorder (*p* = 0.005). We found no statistically significant association between good adherence to CPG’s and lower incidence of events (*p* = 0.853). Clinician adherence to guidelines for secondary prevention of cardiovascular disease was low in the primary care setting.

## 1. Introduction

Cardiovascular disease (CVD) remains the number one cause of death in the general population [1]. Coronary heart disease and stroke account for more than half of all incidences of CVD [2]. People who already have CVD are at the greatest risk of suffering cardiovascular events and death, but effective secondary prevention may reduce the risk of recurrence and improve chances of survival [3,4].

Evidence-based clinical practice guidelines (CPGs) [5,6] recommend pharmaceutical intervention, surgical revascularisation and behavioural changes [3]. CVD morbidity and mortality could be greatly reduced in secondary prevention through more effective behavioural interventions, risk factor control and optimal use of prophylactic treatments [7], which is why healthcare professionals must make every effort to correctly implement clinical practice guidelines.

Previous European studies have investigated adherence to clinical guideline treatment recommendations (EUROASPIRE I-IV [7,8,9]), showing that although these recommendations are increasingly applied in secondary prevention, there is still considerable room for improvement. In 2015, the authors of the EUROASPIRE IV study [9], who obtained data from 7988 heart disease patients aged under 80 years from 24 European countries, found that most patients did not achieve the reference standards for secondary prevention despite the correct use of recommended therapies, because behavioural changes were insufficient. In 2011, another group of researchers conducted a cross-sectional survey of primary care physicians in Spain to measure awareness and implementation of the European CPGs on CVD prevention, finding that although most physicians were familiar with the guidelines, only one third applied them [10]. 

With the aim of improving patient care in secondary prevention, in 2011 our research team assessed the efficacy of a comprehensive programme for secondary cardiovascular prevention in primary care (PREseAP study [11,12]). The programme did not effectively reduce cardiovascular morbidity and mortality, but it did improve some aspects related to healthy habits. A better understanding of the actions taken within healthcare systems—specifically in primary care—and their results could help us to develop solid, evidence-based prevention and care models to address all the difficulties associated with CVD. The aim of this study was to assess clinicians’ adherence to CPGs with regard to recommended treatments in patients with cardiovascular disease in the primary care setting; to determine which factors were associated with good adherence; and to measure the association between these factors and patient prognosis.

## 2. Materials and Methods 

We conducted an ambispective observational cohort study in the primary care setting, with the participation of 21 healthcare centres in eight Spanish regions (Catalonia, Castilla y León, Madrid, the Basque Country, Aragon, the Balearic Islands, Extremadura and the Valencian Community). Participants were selected from the control group of the PREseAP study [11,12]. We included all CVD patients who attended primary care consultations between January 2004 and May 2005 and who met the inclusion criteria. We followed up with them from inclusion until 2009 or until the first cardiovascular event. The inclusion criteria were: men and women aged between 50 and 85 years; being diagnosed with coronary heart disease, stroke and/or peripheral arterial disease between January 2004 and May 2005; possessing a medical history that was included in the electronic health records; and providing written informed consent. The exclusion criteria were: having a severe or terminal disease, being bedridden, having an unstable condition (severe heart valve disease, angina less than 28 days after an acute myocardial infarction, severe ventricular arrhythmias in the previous six months) due to their difficulties in attending the visits, or having subarachnoid haemorrhage or cardioembolic stroke due to known heart valve disease. This study was conducted in accordance with the Declaration of Helsinki and was approved by the institutional ethics committee.

At the end of the follow-up period, we identified the patients who had received the drug therapies recommended in the European CPGs on cardiovascular disease prevention [13] (CPG’s adherent group) and compared them with the patients who had not (CPG’s non-adherent group). In patients with coronary heart disease, recommended drugs included antiplatelet drugs, lipid-lowering drugs, beta-blockers, angiotensin-converting-enzyme inhibitors (ACE-Is) or angiotensin II receptor blockers (ARBs). In patients with stroke or peripheral arterial disease, recommended drugs included antiplatelet or lipid-lowering drugs [2,13].

Our data source was medical histories from primary care consultations and tertiary referral hospitals. The outcome variables were cardiovascular hospital admissions (for acute myocardial infarction, unstable angina, stroke, coronary revascularisation, acute lower limb ischaemia, amputation following ischaemia, vascular surgery), all-cause mortality and cardiovascular mortality. 

We collected the following sociodemographic variables: Age (50 to 59 years, 60 to 69 years, 70 years and older), sex, marital status (single, married, widowed, separated/divorced), work situation (actively employed, retired, unemployed, sick leave/incapacitated, other), level of education (no education, primary education, secondary/tertiary education) and region. We also collected information on smoking and drinking habits, categorising smoking status as never smokers, current smokers (smoked within the previous year) and ex-smokers (quit smoking more than one year ago); and defining heavy drinkers as men having more than 40 g (4 units) of alcohol per day and women having more than 24 g (2.4 units) per day. Lastly, we collected data regarding patients’ medical history (cardiovascular disease, diabetes mellitus, hypertension, dyslipidaemia, chronic obstructive pulmonary disease, kidney failure, psychiatric disorder), physical examination findings (body mass index (BMI), waist circumference, systolic and diastolic blood pressure), lab results (blood glucose, total cholesterol, high-density lipoprotein HDL cholesterol, low-density lipoprotein LDL cholesterol, triglycerides, creatinine), and prescribed drugs (antiplatelet drugs [acetylsalicylic acid, clopidogrel], beta blockers, ACE-Is, ARBs, anticoagulants, lipid-lowering drugs, other antihypertensive drugs such as diuretics, calcium channel blockers or alpha blockers).

At the end of the follow-up, we assessed control of risk factors, defining good control of hypertension as blood pressure below 140/90 mmHg; good control of dyslipidaemia as LDL below 100 mg/dL; good control of diabetes as glycated haemoglobin below 7%, or below 8% in patients with advanced complications; good control of smoking as being a non-smoker; and good control of weight as not being obese (BMI > 30 kg/m^2^), overweight (BMI > 25 kg/m^2^) or centrally obese (waist circumference >102 cm in men and >88 cm in women).

### Statistical Analysis

We calculated the sample size from the number of participants in the control group of the PREseAP study (N = 600). Assuming an incidence of cardiovascular events of 18% in the CPG’s adherent group, and 30% in the CPG’s non-adherent group and accepting a loss due to missing information in 10% of clinical records, the estimated sample size had to be at least 219 patients in each group (438 patients total) to detect differences of 12% with a significance level of 0.05% and a statistical power of 80%. 

We performed a descriptive analysis of the sample, presenting the data by frequency and percentage. To measure the association of adherence to CPG’s and non-adherence to CPG’s with the categorical variables, we used the Chi-square test. To measure the association of adherence to CPG’s with morbidity and with sex, we calculated odds ratios (OR) and their 95% confidence intervals (CIs) using logistic regression. To measure the associations between the explanatory variables and occurrence of cardiovascular events, we used Cox regression models, calculating the hazard ratio (HR) and its 95% CI and adjusting the analysis for each explanatory variable. We fitted a multivariable Cox regression model based on the Akaike information criterion (AIC). The level of statistical significance was set at *p* < 0.05. For all analyses we used IBM SPSS statistics V23.0 (IBM, Armonk, NY, USA).

## 3. Results

Of a total of 600 patients in the control group of the PREseAP study, 438 patients met the inclusion criteria and were included in this study. The remaining patients were excluded due to missing information or could not be located during the follow-up. Table 1 shows the sociodemographic characteristics of the 438 study participants, of whom 72.4% (*n* = 317) were men and 45.4% (*n* = 199) were aged 70 years or older. Of all the participants, 38.6% (*n* = 169) received the drug therapies recommended in the European clinical guidelines (CPG’s adherent group) and 61.4% (*n* = 269) did not (CPG’s non-adherent group). The bivariable analysis did not show an association between adherence to CPG’s and patient characteristics (age, sex, education level, marital status). We only found an association with area of residence, with the lowest adherence to CPG’s in Aragón and the highest in Madrid.

Table 2 shows the distribution of the types of cardiovascular disease, risk factors, and incidence of cardiovascular events and cardiovascular mortality among participants. The most common cardiovascular disease was coronary heart disease (*n* = 162; 37.0%) and the most common risk factor was hypertension (*n* = 294; 67.1%). There were no significant differences in the proportion of patients who received the drug therapies recommended in the CPGs between groups of patients according to cardiovascular disease (*p* = 0.087). Additionally, the proportion of patients who received the drug therapies recommended in the CPGs was higher in patients with hypertension than in those without (43.9% vs. 27.8%; *p* = 0.001); was higher in patients with dyslipidaemia than in those without (50.8% vs. 24.3%; *p* < 0.001); was higher in patients with diabetes than in those without (50.4% vs. 33.7%; *p* = 0.001); and was higher in patients without psychiatric disorder than in those with (40.3% vs. 13.8%; *p* = 0.005). Moreover, we found that 2.3% of patients (*n* = 10) died during follow-up and 18.3% (*n* = 80) were admitted to hospital due to cardiovascular disease, without statistically significant differences between the CPG’s adherent and CPG’s non-adherent groups.

The most commonly prescribed treatment was antiplatelet drugs, used by 88.4% of patients (*n* = 387) (Table 3). And there are significant differences between groups of disease (*p* = 0.009). Table 4 displays the association of adherence to CPG’s with morbidity factors and sex. Patients with stroke were more likely to receive the recommended drug therapies than those with coronary heart disease (*p* = 0.005). Other factors that increased the likelihood of adherence to CPG’s were being diagnosed with hypertension (*p* = 0.040), dyslipidaemia (*p* < 0.001) or diabetes (*p* = 0.025), and not having a psychiatric disorder (*p* = 0.016) (Table 4).

Table 5 shows the association of prognosis, measured by incidence of cardiovascular events (hospital admission or death) with adherence to CPG’s and other factors. We found no statistically significant association between adherence to CPG’s and lower incidence of events (*p* = 0.853). Prognosis was worse in patients aged over 70 years compared with those aged 60 to 69 years (*p* = 0.008), in patients who were incapacitated or on sick leave compared with those who were actively employed (*p* = 0.006), and in patients with diabetes (*p* = 0.014) or depression (*p* = 0.013).

Table 6 shows the association between adherence to guidelines and control of the main cardiovascular risk factors. Prescription of the recommended drugs was associated with good control of LDL cholesterol (*p* = 0.028), but not with good control of hypertension, blood glucose, weight or smoking.

## 4. Discussion

In our study, 38.6% of CVD patients treated in the primary care setting received the drug therapies recommended in the European guidelines on cardiovascular disease prevention in clinical practice. We consider this to be a low proportion, given the efficacy of these drugs in secondary prevention [5], and believe health systems should be aware of this concern. Patients with stroke were more likely to receive the recommended drugs compared with those who had coronary heart disease or peripheral arterial disease. The demographic characteristics, with the exception of region of residence, were not associated with better adherence to CPGs. Having hypertension, dyslipidaemia or diabetes increased the likelihood of adherence. Prescription of the recommended drugs was not a determining factor of prognosis in these patients during the study period, but other factors, such as being older than 70 years, being on sick leave or incapacitated, and having diabetes or depression, were statistically associated with a higher probability of suffering a cardiovascular event. With regard to control of risk factors, adherence to CPGs was significantly associated with good control of LDL cholesterol only.

Other authors also reported low adherence to clinical practice guidelines regarding to recommended treatments in patients with other cardiovascular pathologies. Barnett et al. [14] analysed 9570 patients and found that over a third of patients with atrial fibrillation did not receive the drug therapies recommended in the CPGs. In addition, they found no association between guideline-concordant care and improved risk-adjusted outcomes. On the other hand, Komajda et al. [15] assessed physicians’ adherence to guideline-recommended medications in 6669 patients with heart failure with reduced ejection fraction through a survey. They found that the global adherence score was good in 23% of patients, moderate in 55%, and poor in 22%, and good adherence to drug treatment guidelines was associated with better clinical outcomes during 6-month follow-up.

We did find statistically significant differences (*p* < 0.001) between the prescription of lipid lowering drugs in patients with coronary heart disease (77.2%), stroke (51.1%), peripheral arterial disease (46.7%), with 2 or more disease conditions not including CHD (56.6%) and with 2 or more disease conditions including CHD (75.9%).This may be because there is less evidence of the benefits of lipid lowering treatment in patients with stroke or peripheral arterial disease than in patients with coronary heart disease [16].

In the EUROASPIRE III study [7], conducted from 2006 to 2007, 94.2% of patients with coronary heart disease were treated with antiplatelet drugs, 90.1% with lipid-lowering drugs, 81.6% with beta-blockers and 71.5% with ACE-Is or ARBs. In the present study, these proportions were lower (88.4%, 68.0%, 68.2% and 38.7%, respectively), since other cardiovascular pathologies were included. The 2015 EUROASPIRE IV study [9] reported that medication use in CVD had continued to increase, with 94% of patients using antiplatelet drugs, 86% using lipid-lowering drugs, 83% using beta-blockers and 75% using ACE-Is or ARBs. The authors also found considerable differences in clinical practice between the participating countries. 

In our study, patients who had received the drug therapies recommended in the CPGs had a slightly better prognosis, but this association did not reach statistical significance (Table 6). This group had more pre-existing diseases (hypertension, dyslipidaemia and diabetes) than the group whose treatment did not adhere to CPGs, probably because healthcare professionals tend to take greater care when prescribing treatment to patients with cardiovascular risk factors. As a result, the benefits of the drug therapy may have been “diluted” in these high-risk patients. We would have to conduct a controlled clinical trial to ensure the CPG’s adherent and CPG’s non-adherent groups did not differ in important variables such as prevalence of risk factors. However, this would be unethical, given the proven benefit of prophylactic drugs.

Although the traditional risk factors explain much of the risk of cardiovascular disease, psychological factors have also been shown to predict an adverse result. The psychological factor most frequently studied over the last decade is depression [17]. In our study, patients with depression had a 75% higher cardiovascular risk. During the study period, physicians used Goldberg’s anxiety and depression scale (GADS) [18], as per usual clinical practice, during consultations. A recent scientific statement from the American Heart Association recommends that depression be treated as a risk factor for morbidity and mortality in coronary heart disease, and that patients be routinely screened for this psychological disorder [19].

Age is clearly associated with prognosis in CVD. Normally elderly patients are more frail and have more comorbidities than younger patients, and they are also affected by specifically geriatric conditions. These factors limit treatment options, reduce adherence to treatment and lead to a worse prognosis [20]. In our patients, half of whom were aged over 70 years, there was no association between age and being receiving the recommended drug therapies (Table 5) but we did find an association between older age and poor prognosis (Table 6). Given that the elderly make up a large proportion of chronic cardiovascular disease sufferers, it would be useful to design studies focusing exclusively on this age group [21].

The strengths of our study include its retrospective component, which made it cheaper than a completely prospective study while also enabling us to effectively assess the effect of risk factors on comorbidities by ensuring proper temporality, enabling an analysis of cause and effect. One limitation was the low prescription of recommended drug therapies, which meant we had far fewer patients who received the drug therapies recommended in the CPGs than patients who did not. In addition, although our patients had high cardiovascular risk, having suffered a previous cardiovascular event (secondary prevention), our follow-up period may have been too short to assess the association between recommended drug therapies and prognosis. Follow-up periods in previous studies have been of similar length, however.

Although data were collected between 2004 and 2009, the current recommendations for secondary cardiovascular prevention remain unchanged. Thus, this study allows for a better understanding of associated factors for the implementation of CPGs and may provide key information for health systems. To ensure that evidence-based guidelines are followed, health systems must adopt strategies to facilitate adherence to preventive services guidelines. Further efforts are needed to find the best encouragements to overcome the barriers to implementation.

## 5. Conclusions

Clinician adherence to European CPGs for secondary prevention of cardiovascular disease was low in the primary care setting. Having stroke, being diagnosed with hypertension, dyslipidaemia or diabetes increased the likelihood of receiving the recommended drugs. Adherence to clinical guidelines improved control of LDL cholesterol but did not significantly improve patient prognosis in secondary prevention.

## Figures and Tables

**Table 1:** Participants’ sociodemographic characteristics, by clinician adherence to clinical practice guidelines (CPGs) for secondary prevention of cardiovascular disease.

Characteristic	Total, *n* (%)	Non-Adherence to CPGs, *n* (%)	Adherence to CPGs, *n* (%)	*p* Value ^1^
**Total**	438	269 (61.4)	169 (38.6)	
**Sex**				
man	317 (72.4)	193 (60.9)	124 (39.1)	
woman	121 (27.6)	76 (62.8)	45 (37.2)	0.711
**Age**				
50 to 59 years	118 (26.9)	70 (59.3)	48 (40.7)	
60 to 69 years	121 (27.6)	75 (62.0)	46 (38.0)	
≥70 years	199 (45.4)	124 (62.3)	75 (37.7)	0.860
**Marital status**				
single	19 (4.3)	12 (63.2)	7 (36.8)	
married	348 (79.5)	210 (60.3)	138 (39.7)	
separated/divorced	10 (2.3)	9 (90.0)	1 (10.0)	
widowed	61 (13.9)	38 (62.3)	23 (37.7)	0.301
**Work situation**				
actively employed	67 (15.3)	39 (58.2)	28 (41.8)	
retired	261 (59.6)	164 (62.8)	97 (37.2)	
other	110 (25.1)	66 (60.0)	44 (40.0)	0.739
**Level of education**				
no education	151 (34.5)	95 (62.9)	56 (37.1)	
primary education	178 (40.6)	104 (58.4)	74 (41.6)	
secondary/tertiary education	109 (24.9)	70 (64.2)	39 (35.8)	0.555
**Region of residence**				
Catalonia	113 (25.8)	57 (50.4)	56 (49.6)	
Castilla y León	78 (17.8)	55 (70.5)	23 (29.5)	
Madrid	34 (7.8)	15 (44.1)	19 (55.9)	
Basque Country	37 (8.4)	26 (70.3)	11 (29.7)	
Aragon	17 (3.9)	15 (88.2)	2 (11.8)	
Balearic Islands	72 (16.4)	45 (62.5)	27 (37.5)	
Extremadura	27 (6.2)	19 (70.4)	8 (29.6)	
Valencian Community	60 (13.7)	37 (61.7)	23 (38.3)	0.005

^1^ Chi square test of independence.

**Table 2:** Distribution of risk factors, type of cardiovascular disease and clinical history, by clinician adherence to clinical practice guidelines (CPGs) for secondary prevention of cardiovascular disease.

Variable	Total, *n* (%)	Non-Adherence to CPGs, *n* (%)	Adherence to CPGs, *n* (%)	*p* Value ^1^
**Total**	438	269 (61.4)	169 (38.6)	
**Smoking**				
non-smoker	167 (38.1)	97 (58.1)	70 (41.9)	
ex-smoker	201 (45.9)	130 (64.7)	71 (35.3)	
smoker	70 (16.0)	42 (60.0)	28 (40.0)	0.418
**Heavy drinker**				
no	405 (92.5)	245 (60.5)	160 (39.5)	
yes	33 (7.5)	24 (72.7)	9 (27.3)	0.165
**Cardiovascular disease**				
CHD	162 (37.0)	108 (66.7)	54 (33.3)	
stroke	92 (21.0)	47 (51.1)	45 (48.9)	
PAD	15 (3.4)	8 (53.3)	7 (46.7)	
≥2 combined diseases (not including CHD)	57 (13.0)	32 (56.1)	25 (43.9)	
≥2 combined diseases (including CHD)	112 (25.6)	74 (66.1)	38 (33.9)	0.087
**Obese/overweight**				
no	181 (41.3)	115 (63.5)	66 (36.5)	
yes	257 (58.7)	154 (59.9)	103 (40.1)	0.444
**Hypertension**				
no	144 (32.9)	104 (72.2)	40 (27.8)	
yes	294 (67.1)	165 (56.1)	129 (43.9)	0.001
**Dyslipidaemia**				
no	202 (46.1)	153 (75.7)	49 (24.3)	
yes	236 (53.9)	116 (49.2)	120 (50.8)	<0.001
**Diabetes**				
no	309 (70.5)	205 (66.3)	104 (33.7)	
yes	129 (29.5)	64 (49.6)	65 (50.4)	0.001
**Heart failure**				
no	406 (92.7)	245 (60.3)	161 (39.7)	
yes	32 (7.3)	24 (75.0)	8 (25.0)	0.101
**COPD**				
no	405 (92.5)	246 (60.7)	159 (39.3)	
yes	33 (7.5)	23 (69.7)	10 (30.3)	0.309
**Chronic kidney disease**				
no	417 (95.2)	260 (62.4)	157 (37.6)	
yes	21 (4.8)	9 (42.9)	12 (57.1)	0.073
**Psychiatric disorder**				
no	409 (93.4)	244 (59.7)	165 (40.3)	
yes	29 (6.6)	25 (86.2)	4 (13.8)	0.005
**Depression**				
no	238 (54.3)	147 (61.8)	91 (38.2)	
yes	196 (44.7)	120 (61.2)	76 (38.8)	0.908
**Anxiety**				
no	288 (65.8)	177 (61.5)	111 (38.5)	
yes	147 (33.6)	90 (61.2)	57 (38.8)	0.962
**CV mortality**				
no	428 (97.7)	264 (61.7)	164 (38.3)	
yes	10 (2.3)	5 (50.0)	5 (50.0)	0.453
**CV hospital admission**				
no	358 (81.7)	221 (61.7)	137 (38.3)	
yes	80 (18.3)	48 (60.0)	32 (40.0)	0.774

^1^ Chi square test of independence. CHD: coronary heart disease; PAD: peripheral arterial disease; COPD: chronic obstructive pulmonary disease; CV: cardiovascular.

**Table 3:** Distribution of patients who received the recommended drug therapies by therapeutic group and type of cardiovascular disease.

Therapeutic Group		Cardiovascular Disease					
	Total (*n* = 438)	CHD (*n* = 162)	Stroke (*n* = 92)	PAD (*n* = 15)	≥2 Diseases (Not Including CHD) (*n* = 57)	≥2 Diseases (Including CHD) (*n* = 112)	*p* Value ^1^
	*n* (%)	*n* (%)	*n* (%)	*n* (%)	*n* (%)	*n* (%)	
Anti-platelet drugs	387 (88.4)	155 (95.7)	75 (81.5)	13 (86.7)	48 (84.2)	96 (85.7)	0.009
Lipid-lowering drugs	298 (68.0)	125 (77.2)	47 (51.1)	7 (46.7)	34 (59.6)	85 (75.9)	<0.001
Beta-blockers ^2^	187 (68.2)	117 (72.2)				70 (62.5)	0.089
ACE-Is/ARBs ^2^	106 (38.7)	63 (38.9)				43 (38.4)	0.934

^1^ Chi square test of independence. ^2^ This percentage was calculated with respect to the total of patients with CHD and ≥2 combined diseases including CHD (*n* = 274). CHD: coronary heart disease; PAD: peripheral arterial disease; ACE-Is: angiotensin-converting-enzyme inhibitors; ARBs: Angiotensin II receptor blockers.

**Table 4:** Association between adherence to European clinical practice guidelines (CPGs) for preventing cardiovascular disease and sociodemographic and morbidity factors.

Factors	OR ^1^	95% CI	*p* Value
**Age**	0.99	0.97, 1.02	0.678
**Sex**			
man	1		
woman	1.44	0.88, 2.34	0.146
**Cardiovascular disease**			
CHD	1		
stroke	2.25	1.28, 4.00	0.005
PAD	1.23	0.39, 3.85	0.725
≥2 combined diseases (not including CHD)	1.48	0.75, 2.91	0.256
≥2 combined diseases (including CHD)	0.84	0.48, 1.47	0.538
**Hypertension**			
no	1		
yes	1.66	1.02, 2.68	0.040
**Dyslipidaemia**			
no	1		
yes	3.28	2.09, 5.16	<0.001
**Diabetes**			
no	1		
yes	1.69	1.07, 2.69	0.025
**Psychiatric disorder**			
yes	1		
no	4.00	1.30, 12.34	0.016

^1^ age and sex-adjusted odds ratio. CHD: coronary heart disease; PAD: peripheral arterial disease; 95% CI: 95% confidence interval.

**Table 5:** This Association between prognosis, measured by the incidence of cardiovascular events (cardiovascular hospital admission or death), and adherence to European clinical practice guidelines (CPGs) and other factors during follow-up.

Factors	HR ^1^	95% CI	*p* Value
**Age**			
60–69 years	1		
≤59 years	1.26	0.56, 2.84	0.576
≥70 years	2.21	1.23, 3.96	0.008
**Work**			
actively employed	1		
unemployed	2.72	0.52, 14.26	0.236
sick leave/incapacitated	4.22	1.52, 11.66	0.006
retired	1.91	0.61, 6.00	0.266
other	2.36	0.74,7.55	0.149
**Recommended drugs**			
yes	1		
no	0.96	0.62, 1.48	0.853
**Diabetes**			
no	1		
yes	1.72	1.12, 2.65	0.014
**Depression**			
no	1		
yes	1.75	1.13, 2.80	0.013

^1^ age and sex-adjusted hazard ratio. HR: hazard ratio; 95% CI: 95% confidence interval.

**Table 6:** Control of main cardiovascular risk factors, by clinician adherence to clinical practice guidelines (CPGs) for secondary prevention of cardiovascular disease.

Control of CVR Factors	Total, *n* (%)	Non-Adherence to CPGs, *n* (%)	Adherence to CPGs, *n* (%)	*p* Value ^1^
**Hypertension**				
SBP < 140 and DBP < 90	172 (43.1)	117 (68.0)	55 (32.0)	
SBP ≥ 140 or DBP ≥ 90	227 (56.9)	133 (58.6)	94 (41.4)	0.054
**LDL cholesterol**				
<100	172 (46.0)	95 (55.2)	77 (44.8)	
≥100	202 (54.0)	134 (66.3)	68 (33.7)	0.028
**HDL cholesterol**				
women ≥ 50; men ≥ 40	204 (53.4)	134 (65.7)	70 (34.3)	
women < 50; men < 40	178 (46.6)	102 (57.3)	76 (43.7)	0.093
**HbA1c (in diabetics)**				
<7	90 (74.4)	46 (51.1)	44 (48.9)	
7–8	18 (14.9)	9 (50.0)	9 (50.0)	
>8	13 (10.7)	5 (38.5)	8 (61.5)	0.695
**Obesity**				
BMI < 30	247 (62.8)	157 (63.6)	90 (36.4)	
BMI ≥ 30	146 (37.2)	87 (59.6)	59 (40.4)	0.433
**Waist circumference**				
women < 88; men < 102	69 (17.8)	47 (68.1)	22 (31.9)	
women ≥ 88; men ≥ 102	318 (82.2)	196 (61.6)	122 (38.4)	0.313
**Smoking**				
non-smoker	167 (38.1)	97 (58.1)	70 (41.9)	
ex-smoker	201 (45.9)	130 (64.7)	71 (35.3)	
smoker	70 (16.0)	42 (60.0)	28 (40.0)	0.418

^1^ Chi square test of independence. CVR: cardiovascular risk; SBP: systolic blood pressure; DBP: diastolic blood pressure; LDL: low-density lipoprotein; HDL: high-density lipoprotein; HbA1c: glycated haemoglobin; BMI: body mass index.
